# It is all About the Chase: Neurosteroidogenesis in Male Rats is Driven by Control of Mating Pace

**DOI:** 10.2174/1570159X21666221019114535

**Published:** 2023-05-18

**Authors:** Amy S. Kohtz, Cheryl A. Frye

**Affiliations:** 1 Department of Psychiatry & Human Behavior, Division of Neurobiology & Behavior Research, University of Mississippi Medical Center, 2500 N State Street, Jackson, MS 39216, USA;; 2 Comprehensive Neuropsychological Services, PLLC, 490 Western Avenue, Albany, NY 12203, USA

**Keywords:** Mating, paced-mating, testosterone, 5α-androstane-3α-17β-diol (3α-diol), estradiol, aging

## Abstract

**Background:**

Masculine sexual behaviors are dependent on androstane-derived steroids; however, the modulatory effects of mating, and of mating control, on androstane neurosteroidogenesis remain largely unknown.

**Objective:**

Herein, we investigated the effects of mating control, prior sexual experience, and age on brain region specific neurosteroidogenic responses in male rats.

**Methods:**

Effects of acute sexual experience were tested in naïve male rats that either remained sexually-naïve, were exposed to a standard mating chamber, or were either given control of the mating pace in a standard mating chamber (male control) or mated wherein the female stimulus rat controlled the mating pace in a paced-mating chamber (female control). Aged (10-12 months) sexually responsive male rats were similarly euthanized from the homecage or engaged in male controlled or female controlled mating. All rats were euthanized immediately following exposure conditions for radioimmunoassay of steroids in midbrain, hypothalamus, hippocampus and cortex.

**Results:**

Consummatory sexual behavior in male *vs*. female-controlled mating paradigms was altered by age and prior sexual experience. Male-controlled mating increased androstane neurosteroid metabolism, such that complementary increases in the testosterone (T) metabolite 5α-androstane-3α-17β-diol (3α-diol) in the midbrain and hypothalamus of male rats corresponded to decreases in the prohormone, T. 3α-diol were increased in the hippocampus in response to the context alone, and to a lesser degree in response to mating. Mating diminished neurosteroidogenesis in the cortex. Neurosteroidogenesis was overall reduced in aged male rats compared to naïve controls, however, these effects were more prominent in sexually non-responsive aged male rats.

**Conclusion:**

Extending previous findings, these results indicate differential production of androstane neurosteroids in a mating exposure, age and brain region dependent manner.

## 1 INTRODUCTION

There is abundant literature supporting the notion that circulating androgen steroids during critical periods of development exert organizational effects that ready the brain for responses to hormones and in adulthood, exert activational effects on appetitive and consummatory sexual behavior in males [1-3]. Early studies investigating the organizational effects of prenatal or perinatal androgen/anti-androgen exposure, or administration of testosterone to females during development, show that masculine sexual behavior is dependent upon exposure to androgens and estrogens [4-7]. Administering female neonates testosterone (T) or estradiol (E_2_) reduces sexual receptivity tested in adulthood. Furthermore, in these rodents, proximate injections of T produce male typical mounting responses [5], indicating there are required additional activational effects of steroid hormones in adulthood to facilitate male-typical sexual behavior. In utero exposure to aromatase inhibition enhanced E_2_- and progesterone-primed lordosis (female-typical) sexual behavior in both male and female rats. Furthermore, in gonadectomized rodents, testosterone replacement can restore male sexual behavior to males and induce masculine sexual behavior in females [8-10]. These data implicate androstane steroids in an early organizing role, and proximate activating role, in the masculinization of sexual behavior.

Metabolic clearance, production rates, and diurnal cycling of circulating testosterone decline in aging men [11-13]. The relationship between declining androgens and sexual function is unclear, as aging is associated with decreased psychosexual function in both androgen deficient and aged men with normal range androgens [14]. Engaging in physical fitness and sexual behavior can ameliorate androgen decline associated with aging, implicating a possibility for a “use it or lose it” phenomena [15, 16]. Indeed, maintaining neurosteroidogenesis across the lifespan has been implicated as neuroprotective against neurodegenerative diseases [17]. Thus, circulating androgen steroids and functional effects on behavior, including sexual behaviors, across the lifespan may be linked but do not have a causal relationship in men. However, these studies neither account for changes in neurosteroidogenesis across the lifespan nor do they identify changes in neurosteroidogenesis associated with engagement in mating in aged populations.

Engagement in masculine mating behavior, while dependent on androstane-derived steroids, may also induce androstane steroid production and modulate downstream activity. Indeed, mating increases brain concentrations of testosterone (T) and its 5α-reduced metabolite 5α-androstane-3α-17α-diol (3α-diol) in brain regions that mediate reproductive behavior, such as the midbrain [18] and is associated with neural activity in the mesocorticolimbic circuit, including the medial prefrontal cortex (mPFC), nucleus accumbens (NAc) and midbrain ventral tegmental area (VTA) [19-22]. In males, mating behavior also induces Fos expression in neurons that are immunoreactive for both androgen and estrogen receptors [23], implicating a role for both T and estradiol in the brain to influence masculine mating behavior.

Prior reports in females indicate that neurosteroidogenesis in response to mating may be paradigm dependent; as female control of mating (paced mating) increased serum progesterone and 3α-diol, but male control of mating (standard mating) is less effective [24]. The impact of mating control on neurosteroidogenesis remains to be elucidated in males. Herein, we first extended prior reports on mating-induced neurosteroidogenesis in females by investigating the effects of male or female control of mating pace to elicit engagement in mating (mounting latencies) and androstane neurosteroidogenesis in the midbrain, hypothalamus, cortex, and hippocampus of male rats. We hypothesized that androstane neurosteroidogenesis would be enhanced in the midbrain following engagement in mating. We then investigate the role of mating during aging on lifespan neurosteroid production. We predicted that engagement in mating across the lifespan would decrease aging associated declines in androstane neurosteroidogenesis.

## 2 MATERIALS AND METHODS

### 2.1 Subjects

Subjects were adult males, sexually-naïve (55-60 days of age), adult males sexually experienced (60-65 days of age), and mid-aged males (10-12 months). Long-Evans rats were obtained from our in-house breeding colony. Mid-aged males had a minimum of bi-weekly exposure to a sexually responsive female conspecific since sexual maturity. In experiment 1, naïve adult male rats (n = 9), adult male rats with sexual experience for one week (n = 10), mid-aged male rats that were sexually responsive (SR; n = 8), and mid-aged male rats that were sexually non-responsive (SNR; n = 12) were used to determine neurosteroidogenesis in response to aging and sexual experience. In experiment 2, a separate group of naïve adult male rats used to determine neurosteroidogenesis in response to different mating conditions were separated such that rats were non-tested (n = 9), chamber exposed (n = 9), paced-mated (n = 10), or standard mated (n = 10). Similarly, in experiment 3, a separate group of sexually-responsive mid-aged male rats used to determine neurosteroidogenesis produced by different mating conditions were separated such that rats were non-tested (n = 8), chamber exposed (n = 15), paced-mated (n = 13), or standard mated (n = 11). Experimental rats were housed in two to four per cage (45×24×21 cm), which contained woodchip shavings for bedding, in a temperature-controlled room (21 ± 1°C) in the Life Sciences Laboratory Animal Care Facility at The University at Albany-SUNY. The rats lived in a 12/12-h reversed light cycle (lights off at 8:00 h) with unlimited access to Purina Rodent Chow and tap water in their home cages.

### 2.2 Determination of Sexual Endophenotype in Adult and Mid-aged Males

Adult male rats were given the option to engage in sexual behavior with a responsive female in daily 15-minute sessions for 5 consecutive days once they reached 55 days of age. Males were considered sexually responsive if they showed an ejaculatory series on 3 or more days out of 5. The option to engage in sexual behavior began at 55 days of age and occurred 2-3x per week, extending until 10-12 months (time of testing) in mid-aged rats during sessions with experimental females in other protocols. Rats that consistently exhibited ejaculatory series during these sessions were considered sexually-responsive, whereas those rats that consistently did not engage in an ejaculatory series (including a lack of mounts and intromissions) were considered sexually-non responsive. This was confirmed for 5 days prior to testing with daily sessions, as described above, for adult males. In both groups, the last mating experience occurred 3 days prior to exposure for testing. It is of note that mid-aged SNR rats showed an absence of ano-genital investigation, mounts, and intromissions, indicating a lack of sexual motivation.

### 2.3 Male-controlled (Standard) Mating

The standard mating paradigm is a sexual behavior task in which male rats control the pacing of sexual contacts. Rats were placed in the white polycarbonate standard mating chamber (37.5x75x30 cm) for 10 minutes with a hormone-primed receptive female and the latency to mount was recorded to confirm mating behavior.

### 2.4 Female-controlled (Paced) Mating

The paced mating chamber is a sexual behavior task in which the female rat has control over the pacing of sexual contact. The box (37.5x75x30 cm) is white laminate with a clear Plexiglas divider that has a small hole in the bottom, which only the female can pass through. Rats were placed in the paced-mating chamber for 10 minutes with a hormone-primed receptive female, and the latency to mount was recorded to confirm mating behavior. Stimulus females used for sex testing were ovariectomized and implanted with silastic capsules (0.062 i.d., 0.125 o.d.; 10 mm/100 g body weight; Steraloids, Inc., Newport, RI) containing 0.8 mg of 17β-E2 and 40 mg of crystalline progesterone. Implants were retained until sexual receptivity ceased.

### 2.5 Tissue Collection

Immediately after testing, rats were rapidly decapitated and whole brains were collected and stored on dry ice. Whole brains were stored at -80°C for later radioimmunoassay to determine T, E_2_, and 3α-diol levels. At the time of measurement, the hippocampus, hypothalamus, cortex, and midbrain were grossly dissected from whole brains that had been gently thawed on ice per previously reported methods [25].

### 2.6 Radioimmunoassay

T, E_2_, and 3α-diol were measured with radioimmunoassay techniques previously described in detail [18, 26]. Steroids were extracted from brain tissue (homogenized with a glass/Teflon homogenizer in distilled water) with diethyl ether and trace amounts of 3H 3α-diol (purchased from New England Nuclear, Boston, MA). T antibody (T3-125; Endocrine Sciences, Calabasas Hills, CA) was diluted 1:20, 000 and bound between 60% and 65% of (^3^H) T (NET-387: specific activity = 51.0 ci/mmol). The E_2_ antibody (Dr. Niswender, #244, Colorado State University, Fort Collins, CO) was diluted 1:30, 000 and bound approximately 90% of (^3^H) E_2_ (NET-317: specific activity = 51.3 ci/mmol). The antibody for 3α-diol (X-144, Dr. P.N. Rao, Southwest Foundation for Biomedical Research, San Antonio, TX) is highly specific to 3α-diol [27]. The 1:20,000 dilution of this antibody binds 96% of (3H) 3α-diol (NET-806: specific activity = 41.00 Ci/mmol). All standard curves were prepared in duplicate (range = 50 pg-2000 pg). The standards were added to the phosphate assay buffer, followed by the addition of the appropriate antibody and (^3^H) steroid and incubated overnight at 4^o^C. Separation of bound and free steroids occurred by the addition of dextran-coated charcoal. Following incubation with charcoal, samples were centrifuged at 3000×g. The supernatant was pipetted into a glass scintillation vial with a scintillation cocktail. Sample tube concentrations were calculated using the logit-log method of [28], interpolation of the standards and correction for recovery. The intra- and inter-assay coefficients of variance T = 0.09 and 0.09, 3α-diol = 0.09 and 0.11, E_2_ = 0.09 and 0.09, respectively.

## 3 EXPERIMENTAL

### 3.1 Experimental Paradigms

To determine neurosteroidogenesis in response to mating, naïve, adult, and mid-aged male rats were either exposed to the paced-mating chamber alone for 10 minutes, placed in the paced-mating chamber with a receptive female for 10 minutes, placed in the standard mating chamber with a receptive female for 10 minutes, or removed directly from the home-cage, and euthanized. Using 10 minutes instead of 15 minutes produced differences in ejaculation success between mating paradigms in the naïve rats, an effect that may not have been observed had the time been extended to 15 minutes. As such, instead of measuring latencies to ejaculation or the number of ejaculations, we analyzed only the latencies to sexual contact, ano-genital investigation, mounts, intromissions (combined in the analysis) and whether an ejaculation did or did not occur. Brains were collected as described in *Tissue Collection*, and radioimmunoassay was performed to determine steroid levels.

### 3.2 Statistical Analyses

One-way analyses of variance (ANOVAs) were used to determine the effects of sexual experience or age on T, 3α-diol, and E_2_ in the midbrain, hypothalamus, cortex, and hippocampus of male rats. One-way ANOVAs were also used to examine the effects of type of mating stimulation or exposure condition (naïve control, standard mating chamber exposed, standard mated, or paced-mated) on T, 3α-diol, and E_2_ in the midbrain, hypothalamus, cortex, and hippocampus of naïve or mid-aged male rats. Main effects were followed with Bonferroni post-hoc tests to determine group differences. In analyses that failed the Brown-Forsythe test of equal variances, non-parametric one-way ANOVAs using the Kruskal-Wallis f-test were performed instead where indicated with KW before reporting the f statistic. In non-parametric statistics, Dunn’s correction was applied for multiple comparisons. To determine directional shifts in 5α-reduced or aromatized metabolites of T in naïve rats, values for T, E_2_, and 3α-diol were normalized across the entire sample set to account for large variations in normal circulating levels of these steroids ((X-Xmin)/Xmax-Xmin)). Ratios (E_2_:T and 3α-diol:T) were computed for each rat on the normalized data. The normalized ratios of E_2_:T and 3α-diol:T were computed and transformed such that negative values indicated a shift in metabolism towards E_2_, and positive values indicated a shift in metabolisms towards 3α-diol in each rat.

## 4 RESULTS

### 4.1 Mating Success in Male or Female-controlled Mating Paradigms Shifts with Age and with Prior Mating Experience

**Fig. (1):**
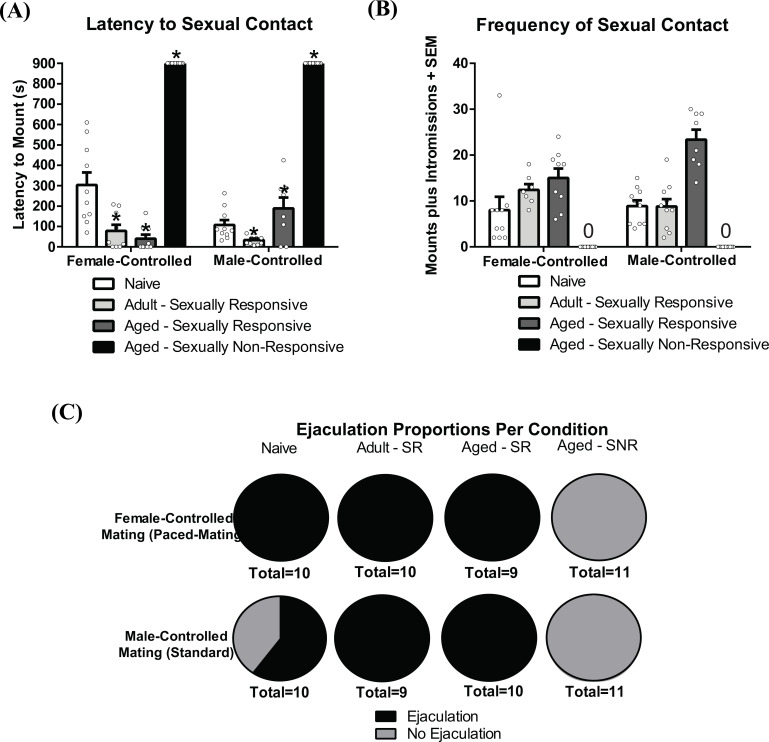
Age and prior sexual experience influence sexual performance in female-controlled (paced) compared to male-controlled (standard) mating. Rats were naïve adult males with no prior sexual experience, adult males with one week of sexual experience, or mid-aged (10-12 months old) sexually responsive to a female, or sexually non-responsive to a female. (**A**) Latencies to mount in female-controlled mating decreased with age and prior sexual experience. (**B**) Age, and mating control interacted to influence the frequency of sexual contacts. (**C**) Proportions of ejaculations observed in each testing condition. As each mating session examined a singular ejaculatory series, statistics on the frequency of ejaculation were not performed. * indicates p < 0.05 in post-hoc t-tests comparing sexual responsivity to naïve controls within mating condition.

Latencies to mount were recorded in each mating paradigm as engagement in consummatory sexual behavior. Mid-aged, sexually non-responsive males did not engage in mating behavior independent of the mating paradigm. There was a main effect of mating condition to influence latencies to mount under both male- and female-controlled mating paces (F_3,70_ = 346.2, *p* < 0.05; Fig. **1A**). As expected, prior sexual experience conferred shorter latencies to mount. However, there was also an interaction between mating control (female-controlled mating, male-controlled mating) and prior mating experience (Naive, adult sexually responsive, aged sexually responsive, aged sexually non-responsive), such that experience influenced consummatory behavior in both male- and female- controlled mating paradigms (F_3,68_ = 9.948, *p* < 0.05; Fig. **1A**). Naïve (virgin) male rats had longer latencies to mount females when females controlled the mating pace as opposed to when the male subject controlled the mating pace (t = 2.969, df = 18, *p* < 0.05). Notably, this latency significantly decreased with mating experience, such that adult sexually-responsive males showed decreased latencies to mount in the female-controlled paradigm compared to naïve rats and mid-aged sexually-responsive male rats showed even lower latencies in the female-controlled paradigm. In addition, mid-aged, sexually-responsive males show a notable increase in latencies to mount when male subjects were in control of the mating pace compared to their adult sexually-responsive counterparts. We further analyzed the frequencies of sexual contact and found a significant interaction between mating control and prior mating experience (F_3,68_ = 4.101, *p* < 0.05; Fig. **1B**). While mating control did not influence the frequency of sexual contact in younger rats, aged-male rats had higher mount/intromission frequencies when they controlled the pace of sexual contact (t = 2.791, df=15, *p* < 0.05). Sexual experience increased the likelihood of observing a full ejaculatory series (Fig. **1C**).

### 4.2 Male-controlled Mating Increased Neurosteroidogenesis and Shifted Androgen Metabolite Ratios Towards 3α-diol, whereas Female-controlled Mating Decreased Neurosteroidogenesis and Shifted Metabolite Ratios Towards E2 of Sexually-naïve Male Rats

**Fig. (2):**
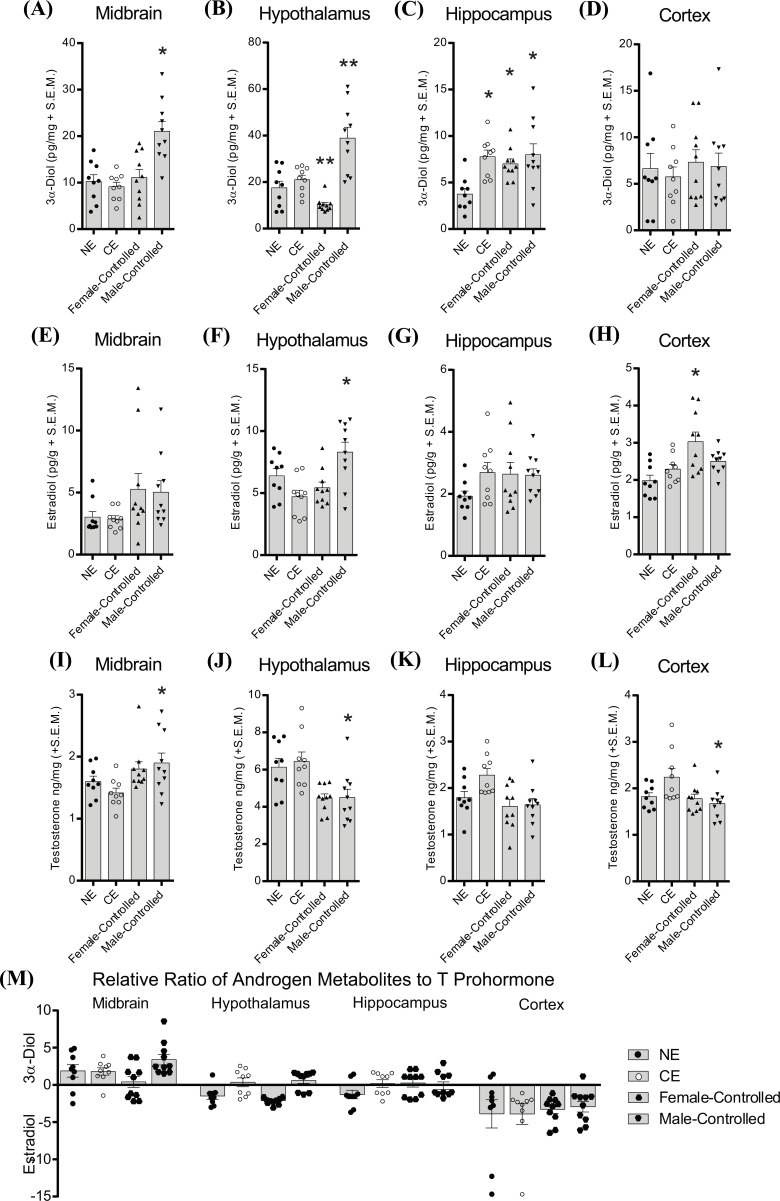
Male-controlled (Standard) mating compared to female-controlled (paced) mating or no mating increased T and 3α-diol in the midbrain, and decreased T and 3α-diol in the hypothalamus of naïve rats. Rats were naïve adult males with no prior sexual experience euthanized from the homecage (no exposure, NE), following 15-min exposure to the standard mating chamber (chamber exposed, CE), following 15-min of female-controlled (paced) mating with a sexually responsive female conspecific (Female-Controlled), or following 15-min of male-controlled (standard) mating with a sexually responsive female conspecific (Male-Controlled). (**A-D**) 3α-diol. (**E-H**) E_2_ in the midbrain (**E**), hypothalamus (**F**), hippocampus (**G**) and cortex (**H**). (**I-L**) T in the midbrain (**I)**, hypothalamus (**J**), hippocampus (**K**), and cortex (**L**). (**M**) Relative metabolism of androgens. *indicates significant differences using post-hoc t tests from non-mated (NE and/or CE) controls (*p* < 0.05). **indicates significant post-hoc f-test differences from all other conditions (*p* < 0.05).

When the male test subject controlled the mating pace, the midbrain (F_3,37_ = 11.39, *p* < 0.05; Fig. **2A**) and hypothalamus 3α-diol (KW: F_4,38_ = 21.94, *p* < 0.05; Fig. **2B**) were increased, compared to those that were not mated, chamber exposed, or paced-mated. In fact, female control of the mating pace decreased hypothalamus 3α-diol (t = 6.95, *p* < 0.05; Fig. **2B**) compared to when males controlled the mating pace. Exposure to the context or to either mating paradigm increased hippocampus 3α-diol (F_3,37_ = 6.53, *p* < 0.05; Fig. **2C**) in naïve male rats, likely attributable to engagement in novelty. There were no significant effects of mating pace on cortex 3α-diol (Fig. **2D**). There were no effects of mating pace on E_2_ in the midbrain (Fig. **2E**) or hypothalamus (Fig. **2F**). In sexually naïve rats, male control of mating pace increased hypothalamic E_2_ (F_3,37_ = 6.69, *p* < 0.05; Fig. **2G**), whereas female control of mating pace increased cortex E_2_ (KW: F_4,38_ = 10.84, *p* < 0.05; Fig. **2H**). Mating enhanced T levels in the midbrain (F_3,37_ = 3.234, *p* < 0.05; Fig. **2I**) when males controlled the mating pace (standard mating), and decreased T levels in the hypothalamus (F_3,34_ = 16.19, *p* < 0.05; Fig. **2J**) and cortex (F_3,34_ = 6.427, *p* < 0.05; Fig. **2L**) irrespective of mating condition, in sexually-naïve rats. Metabolite ratios were shifted in the midbrain and hypothalamus by mating paradigm; wherein female control of mating pace shifted androgen metabolite ratios towards E_2_, whereas male control of mating pace shifted androgen metabolite ratios towards 3α-diol, in the midbrain (F_3,37_ = 3.13, *p* < 0.05), and hypothalamus (KW: F_4,38_ = 19.68, *p* < 0.05; Fig. **2M**), as similarly reflected by increased and decreased 3α-diol in each region, respectively.

### 4.3 Androstane Neurosteroidogenesis in the Midbrain and Hypothalamus are Particularly Sensitive to Aging in Male Rats

**Fig. (3):**
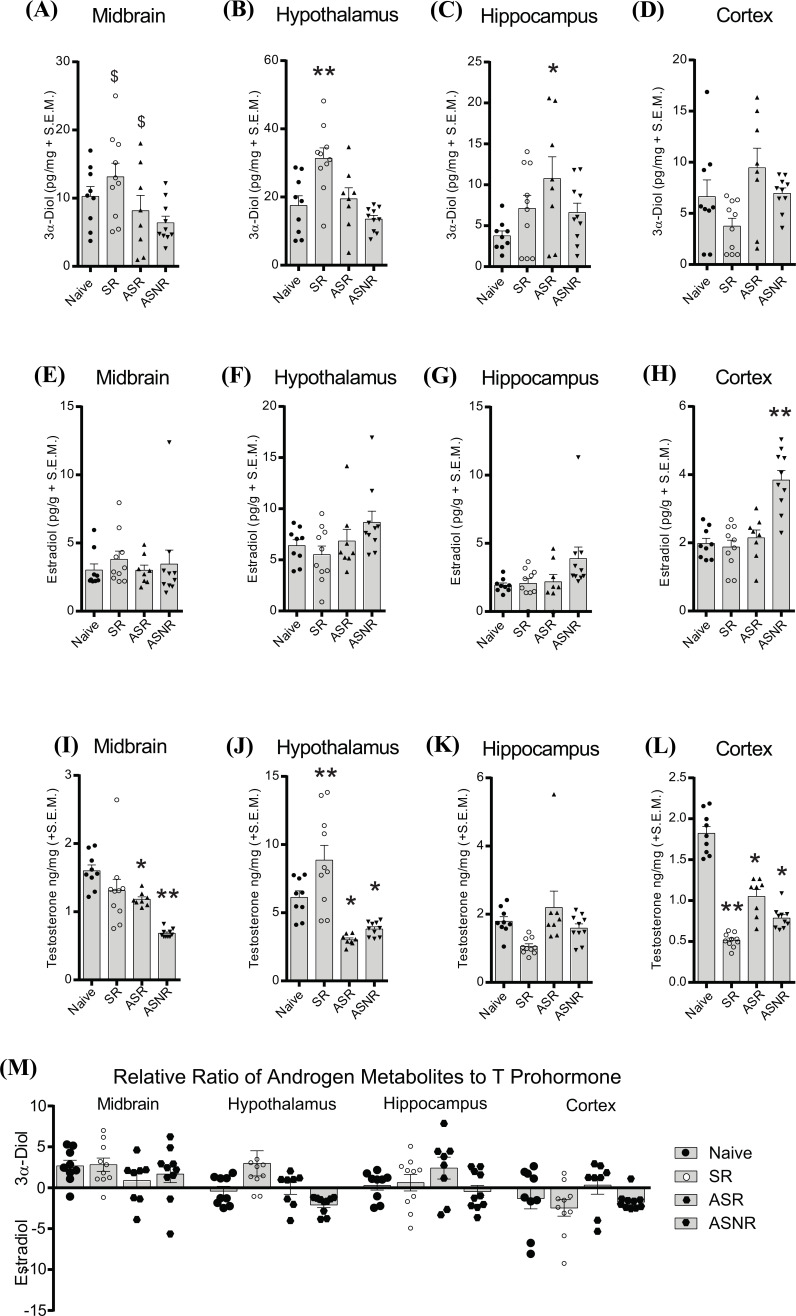
Effects of Aging and Sexual Proclivity on Neurosteroidogenesis. Rats were naïve adult males with no prior sexual experience (Naïve), adult males with one week of sexual experience (adult sexually-responsive, SR), or mid-aged (10-12 months old) sexually responsive to a female (mid-aged sexually-responsive (ASR), or sexually non-responsive to a female (mid-aged sexually non-responsive, ASNR). All rats were euthanized directly from the homecage. (**A-D**) 3α-diol. (**E-H**) E_2_ in the midbrain (**E**), hypothalamus (**F**), hippocampus (**G**) and cortex (**H**). (**I-L**) T in the midbrain (**I**), hypothalamus (**J**), hippocampus (**K**), and cortex (**L**). (**M**) Relative metabolism of androgens. *indicates significant differences using post-hoc t-tests from naïve controls (*p* < 0.05). **indicates significant differences using post-hoc f-tests from all other mating conditions. $ indicates significant differences from mid-aged sexually non-responsive males only.

Midbrain (F_3,36_ = 3.10, *p* < 0.05; Fig. **3A**) 3α-diol levels were increased in high sexual proclivity males (adult or mid-aged) compared to non-sexually responsive mid-aged males. Hypothalamus (F_3,36_ = 8.88, *p* < 0.05; Fig. **3B**) 3α-diol levels were increased as a function of youth and sexual responsivity, such that adult sexually responsive males had greater 3α-diol in the hypothalamus compared to all other groups. Sexually responsive mid-aged male rats had significantly greater hippocampus 3α-diol than did naïve rats (F_3,36_ = 2.98, *p* < 0.05; Fig. **3C**). Sexually non-responsive mid-aged male rats had significantly greater cortex E_2_ compared to all other groups (F_3,36_ =18.18, *p* < 0.05; Fig. **3H**). Similar to 3α-diol, midbrain (KW: F_4,37_ = 25.84, *p* < 0.05; Fig. **3I**) and hypothalamus (KW: F_4,37_ = 27.97, *p* < 0.05; Fig. **3J**) T were increased by sexual experience and decreased by aging. Sexual non-responsivity in mid-aged rats shifted androgen metabolite ratios towards E_2_ in the hypothalamus (KW: F_4,37_ = 16.89, *p* < 0.05; Fig. **3M**).

**Fig. (4):**
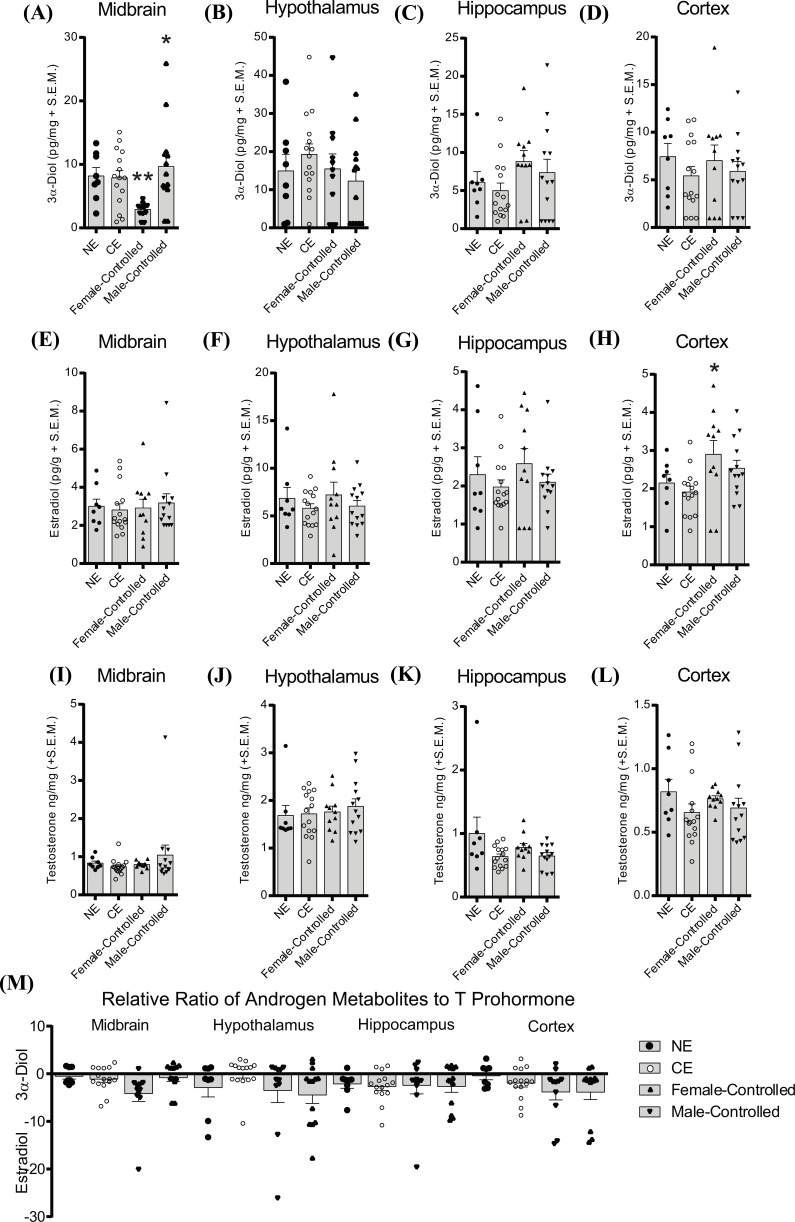
Effects of Mating Condition on Neurosteroidogenesis in Mid-Aged Male Rats. Rats were mid-aged sexually-responsive adult males, and euthanized from the homecage (no exposure, NE), following 15-min exposure to the standard mating chamber (chamber exposed, CE), following 15-min of female-controlled (paced) mating with a sexually responsive female conspecific (Female-Controlled), or following 15-min of male-controlled (standard) mating with a sexually responsive female conspecific (Male-Controlled). (**A-D**) 3α-diol. (**E-H**) E_2_ in the midbrain (**E**), hypothalamus (**F**), hippocampus (**G**) and cortex (**H**). (**I-L**) T in the midbrain (**I**), hypothalamus (**J**), hippocampus (K), and cortex (L).” (**M**) Relative metabolism of androgens. *indicates significant differences using post-hoc t tests from no exposure and/or chamber exposed controls (*p* < 0.05).

Similar to sexually naïve male rats, mid-aged male rats that engaged in female-controlled mating had decreased midbrain 3α-diol levels, whereas males that controlled the mating pace had increased levels of midbrain 3α-diol (KW: F_4,47_ = 12.52, *p* < 0.05; Fig. **4A**), and E_2_ in the cortex was increased when females controlled the mating pace, but not when males controlled the mating pace, compared to chamber exposed controls (F_3,46_ = 3.41, *p* < 0.05; Fig. **4H**). No other effects of the mating paradigm in mid-aged male rats were observed (Fig. **4A-M**).

## 5 DISCUSSION

Our central hypothesis that mating would influence the central production of androgens dependent on male or female control of mating pace was upheld. First, we observed paradigm by age-dependent differences in latencies to mount wherein mount latencies were greater when females controlled the pace for virgin male rats; and mount latencies were diminished when females controlled the pace in mid-aged male rats with a high sexual proclivity. Second, we observed mating-dependent effects on neurosteroidogenesis independent of chamber exposure conditions in the midbrain and hypothalamus of virgin male rats. In these more traditional sex and reward mediating brain regions, we observe brain region dependent responses to engagement in sexual behavior. Hypothalamic T was decreased in response to either mating paradigm, whereas midbrain T and 3α-diol were increased, but only when males controlled the mating pace. We also observed interesting effects of chamber exposure on the prohormones T and E_2_ in virgin males. In cognitive brain regions, such as the hippocampus and cortex, context (chamber) exposure enhanced both T and E_2_ levels. As well, cortex E_2_ was increased by female-controlled mating in virgin males. Similar effects were observed in sexually responsive mid-aged male rats, wherein female-controlled mating increased cortex E_2_, although overall effects of the mating paradigm on neurosteroidogenesis in mid-aged sexually responsive male rats were minimal. Finally, these data show substantial effects of aging on androgen neurosteroidogenesis that may be predicted by sexual proclivity. Mid-aged male rats with low sexual proclivity had greater levels of prohormone E_2_ in the hippocampus and cortex compared to naïve or sexually-responsive rats. High sexual proclivity in mid-aged males increased cortex T and 3α-diol. These data indicate that the profile of neurosteroidogenesis in response to mating may be influenced by the context in which mating occurs, as well as the prior experience or age of the individual.

Many studies have implicated circulating T in appetitive and consummatory male sexual behavior exposure [4-7] and androgen-dependent effects on dopamine neurons and signaling in the midbrain VTA and NAc during adolescence and development [29-31], and adulthood. In adult males, VTA and NAc firing is androgen sensitive, as are the projection neurons from the frontal cortex [32]. Mating, which we show herein to produce androstane neurosteroidogenesis, induces Fos expression in NAc and VTA [22], as does intracerebroventricular administration of T in hamsters [33]. Our data indicate that midbrain T is increased in response to mating paradigms, but only when the male controls the pace (standard mating). These data indicate that male control of sexual pacing may engage more signaling in brain regions that mediate reward, compared to paradigms that involve female control (paced-mating), in male rats.

It is intriguing that we observed decreased T levels in the hypothalamus following engagement in mating in naïve male rats. Unlike the midbrain VTA, T has negative effects on dopamine release in the NAc, wherein administration of high doses of T intracerebroventricularly decreases NAc dopamine release [34]. Blocking dopamine receptors in the NAc can result in decreased anticipatory level changes in search of a female [35], indicating that dopamine in the NAc influences motivation to acquire a female conspecific. As such, the decrease we observed in hypothalamic T may also indicate enhanced dopamine mediated motivation to access the female conspecific.

Androgen activity in dopamine cell bodies of the VTA may be strongly implicated in reward action associated with mating stimuli. Reciprocal inputs between midbrain dopamine neurons and lateral hypothalamic neurons [36] involved in novelty detection [37] are heavily implicated in reward determination, reinforcing the learning of novel stimuli and contributing to the differentiation between the novel and non-novel stimuli in rats [38]. Our results implicate a clear distinction between naturalistic mating paradigms (*e.g*., female-controlled paced mating) and experimentally normative mating paradigms (*e.g*., male-controlled, standard mating). In the case of testing male rodents, female-controlled mating paradigms can be considered administering sexual reward intermittently, whereas male-controlled mating paradigms present free access to reward (receptive female). Mating can be used in males as an unconditioned stimulus to elicit learned behaviors in non-rodent animal models. In male Japanese quail, mating can be used as an unconditioned stimulus to promote sign tracking behavior towards the conditioned stimulus [39]. Male quail that learn conditioned signals for reproductive opportunities have increased reproductive success compared to those that do not [40]. These data replicate strategies used during drug-seeking and acquisition; thus, we speculate that intermittent *versus* free access to sexual *via* the use of different paradigms may induce different neurobiochemical results indicative of changes in strategy to acquire a reward. Strategies for reward acquisition may be influenced by neurosteroidogenesis in the hippocampus and cortex, as we report herein. One limitation of the current study is that we did not perform more detailed analyses of the approach and consummatory behaviors observed during mating in each of the paradigms. These data, in particular the inter-mount-interval as a measure of pacing, would have greatly improved the ability to interpret our results in the context of reward acquisition strategies.

In addition to T’s effects at intracellular cognate androgen receptors [41], some effects of androgens on cognitive function may be mediated by the T metabolite, 3α-diol [42, 43]. Notably, unlike T, which binds with high affinity to androgen receptors, 3α-diol binds with greater affinity to estrogen receptor beta (ERβ; [44]) and GABA/benzodiazepine receptors (GBRs; [45]). Many anti-androgenic drugs, which act by inhibiting formation of 3α-diol, have side effects, which include a detriment to sexual function and cognition [46, 47]. Interestingly, we show that the direction of 3α-diol in response to naïve mating is congruent with T in the midbrain, hypothalamus, and hippocampus. Conversely, in cortex, 3α-diol appears to act independently of T. GABA action in the cortex strongly influences cognitive flexibility, a function that can also be enhanced by prior sexual experience [48, 49]. Together, these data indicate that 3α-diol activity in the cortex may act independent of androgen receptor activation to modulate cognitive flexibility in response to mating.

## CONCLUSION

Prior reports indicate that the effect of copulatory behavior to induce circulating testosterone is rapidly habituated. Our results support these conclusions, and extend them, such that mid-aged male rats with long-term sexual experience do not produce T in the brain in response to mating stimuli, however, T metabolites are altered in select brain regions, *e.g*., midbrain 3α-diol and cortical E_2_. Our data indicate that the type of mating stimuli, *e.g*., female control *vs*. male control, strongly influences the degree of neurosteroidogenesis in reward- and cognition-associated brain regions in naïve, but not sexually experienced, male rats. Notably, the endogenous status of rats (*e.g*., prior sexual experience, age) and the mating paradigm used both strongly influenced the direction and location of neurosteroid effects. The endogenous status of rats (prior sexual experience and proclivity, age) greatly impacted the patterns of neurosteroid responses when compared between male and female-controlled mating. This pattern of results indicates that acute neurosteroid effects of sex exposure may habituate over time and exposures, with the exception of the midbrain, but surprisingly not hypothalamic, 3α-diol. These data raise important questions regarding the nature of steroidogenesis during novel reward-relevant stimuli. Given the localization of induction (*e.g*., midbrain and hypothalamus *versus* cortex and hippocampus) androgen neurosteroidogenesis and the previously identified multitude of roles of androgen steroids in modulating dopamine signaling therein; androgen neurosteroidogenesis may play a facilitating role in dopamine signal transduction to assist in encoding towards reward stimuli, or novelty and contexts, in a brain region dependent manner. Future studies on the direct role of androgens during mating or cognitive stimuli on dopaminergic signaling will elucidate mechanisms in these complex interactions.

## Data Availability

The data supporting the findings of this article are available upon request from the corresponding author.

## LIST OF ABBREVIATIONS

3α-diol: 5α-androstane-3α-17β-diol
CE: Chamber Exposed
E_2_: Estradiol
ERβ: Estrogen Receptor Beta
GBRs: GABA/Benzodiazepine Receptors
KW: Kruskal-Wallis
mPFC: Medial Prefrontal Cortex
ASR: Mid-Aged Sexually Responsive
ASNR: Mid-Aged Sexually Non-Responsive
NE: No Exposure
NAc: Nucleus Accumbens
SNR: Sexually Non-Responsive
SR: Sexually Responsive
T: Testosterone
VTA: Ventral Tegmental Area
